# Facile Fabrication of Silk Fibroin/Off-Stoichiometry Thiol-Ene (OSTE) Microneedle Array Patches

**DOI:** 10.3390/mi14020388

**Published:** 2023-02-04

**Authors:** Yuqian Yang, Zhiqing Xiao, Lexin Sun, Zitao Feng, Zejingqiu Chen, Weijin Guo

**Affiliations:** 1 Department of Biomedical Engineering, Shantou University, Shantou 515063, China; 2 Department of Biomedical Engineering, University of Michigan, Ann Arbor, MI 48109, USA; 3 Department of Biology, Shantou University, Shantou 515063, China

**Keywords:** microneedle, silk fibroin, drug delivery, off-stoichiometry thiol-ene, OSTE, insulin, replica molding

## Abstract

Microneedles have been used in various applications in biomedical engineering, including drug delivery, biosensing, and vaccine delivery. In this study, we develop a novel protocol to fabricate silk fibroin/off-stoichiometry thiol-ene (OSTE) hybrid microneedle array patches. Silk fibroin, as a natural biomaterial, has been proven to be suitable as a drug carrier. Firstly, drug (we use insulin in this experiment) dissolved in silk fibroin solution is deposited on a microneedle mold and dried thoroughly. After that, silk fibroin needle tips are formed on the OSTE base by replica molding. We investigated the influence of the silk fibroin concentration on the length of silk needle tips and found that the silk concentration had a small influence on the tip length. We also tested the mechanical strength of the microneedles by inserting them into gelatin gel for dummy drug delivery tests. Such composite structures have the potential to increase the delivery efficiency by delivering the whole silk tip into the dermis.

## 1. Introduction

Hypodermic needle-based injection, as one of the most widely used methods for drug delivery in the world, guarantees the effective delivery of drugs but comes with the problems of invasiveness, requiring professional administering skills, and generating sharp waste. Transdermal drug delivery overcomes the drawbacks of hypodermic needle-based drug delivery and is advantageous for site-specific and controlled drug delivery. As a result of the need of the successful delivery of larger molecules across skin, the hybrid between these two conventional delivery methods is under development [1]. A microneedle is a sharp needle on a micrometer scale that can penetrate the epidermis of the skin, reach the dermis, and release drugs in a painless and minimally invasive way. To ensure the rigidity of the microneedles, metal and silicon have been used to fabricate microneedle array patches [2,3]. However, they are expensive and increase the burden of disposing of biohazardous waste. Some synthetic polymers are being developed to replace them, such as polylactic-co-glycolic acid (PLGA), poly-L-lactide (PLA), and polyvinyl alcohol (PVA) [4,5]. However, the high temperature needed for melting such materials may lead to the ineffectiveness of the carried drugs such as insulin. Silk fibroin is a natural biomaterial from *Bombyx mori*. It has been proven to be suitable as a drug carrier used in microneedles because of its advantageous features. When it dissolves in our skin, it is not harmful or toxic and will not generate any inflammatory degradation products [6]. Furthermore, it can help improve the mechanical strength of microneedles due to its high structural rigidity [7]. Remarkably, the drug activity can last for a long time when using fibroin protein as a carrier [8,9]. Apart from these, its high-water solubility can guarantee the complete dissolution of drugs without the need for high temperatures or other solvents, and rapid dissolution once inserted into the skin. Furthermore, it is widely available and cheap [10]. Recently, more studies used silk fibroin as a microneedle matrix, but pure silk fibroin microneedles or silk fibroin base may lead to burst release of drugs, which still causes problems of drug build-up [11,12]. Moreover, pure silk fibroin microneedle array patches may waste drugs since only the microneedle tips penetrating the skin can release drugs. The drugs left in the flat base or the microneedle bottoms may not be absorbed by the skin. In addition, present methods of fabricating silk fibroin microneedles need a lot of complex operations, including water vapor annealing, lyophilization, and centrifugation [13,14,15]. Off-stoichiometry thiol-ene (OSTE) is a novel polymer which is photocurable and enables rapid prototyping of microstructures [16]. Its tunable mechanical properties may help to form a microneedle array patch with suitable rigidity that not only can penetrate into skin but also conformably wrap around the skin surface by adjusting the off-stoichiometric ratio alone. Based on the working mechanism of drug delivery, microneedles are categorized into five types, including solid, coated, hollow, dissolving, and hydrogel-forming microneedles [17]. Among them, dissolving microneedles have some significant advantages in biodegradable components, ease of manufacturing, and one-step application [1]. In this study, we develop a protocol for facile fabrication of microneedles consisting of silk fibroin tips and an OSTE base to ensure mechanical strength and simplify the fabrication procedures. We deposit the silk fibroin solution with dummy drugs (insulin in this work) on the microneedle mold to form the tips and cure OSTE on them to form the base, which only needs the conditions of room temperature and normal ambient light. The microneedle array patches fabricated by this method are expected to make the whole silk fibroin tip penetrate into the dermis and improve the delivery efficiency of drugs. More importantly, the protocol is suitable for large-scale manufacturing and may find immediate applications in the market of microneedles for drug delivery.

## 2. Materials and Methods

A round microneedle mold (for 385 needles) with a diameter of 17.5 mm, tip distance of 700 μm, needle diameter of 270 μm and needle height of 500 μm is obtained from Engineering for Life (EFL-MMN-500, Suzhou, China). Novo Nordisk (0.454%, Tianjin, China) provides the insulin solution. Silk fibroin is from Shandong Qilu Biotechnology Group (Liaocheng, China). Food dye is from Lianyungang Xinai Food Technology Company (Lianyungang, China). OSTE is prepared according to a previous report [18]. Gelatin is from Shanghai Macklin Biochemical Co., Ltd. (Shanghai, China).

### 2.1. Fabrication Procedures of Microneedle Array Patches

**Figure 1:**
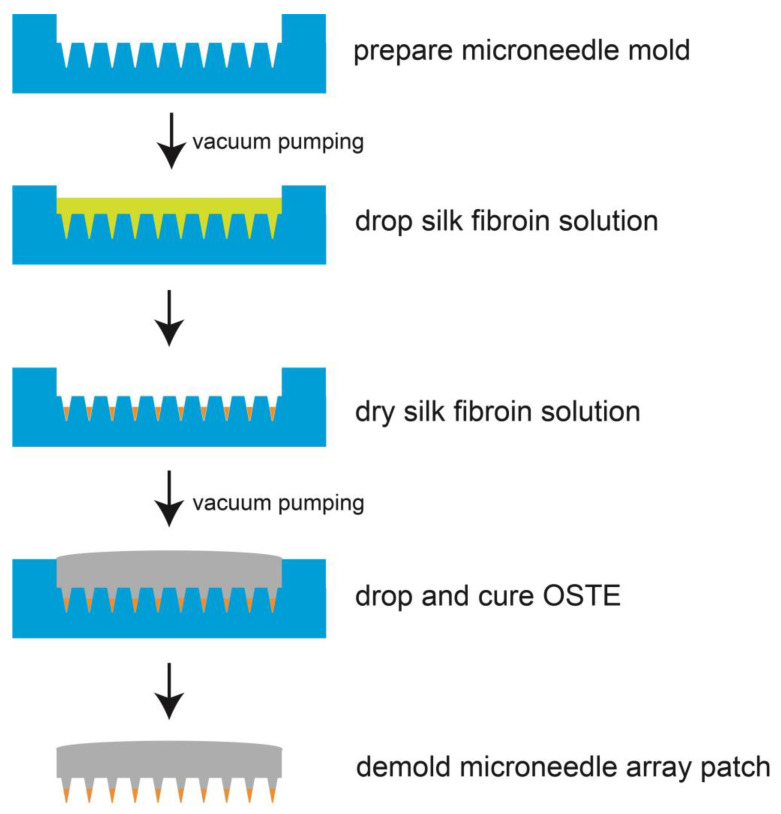
The schematic of the fabrication of microneedle array patches. At first, we prepare the mold, then we drop silk fibroin solution on the mold and make the silk fibroin solution fills the mold. After the silk fibroin solution is fully dry on the mold, we drop OSTE onto it and cure the OSTE. Lastly, we demold the OSTE microneedle array patch from the mold.

For the fabrication procedures, first we prepared the silk fibroin solution with insulin by mixing insulin solution (weight ratio as 33.98%), DI water (weight ratio as 50.98%), purple food dye solution (weight ratio as 8.50%) and silk fibroin (weight ratio as 6.54%). We assume that the densities of insulin solution, DI water, and dye solution are 1.0 g/cm^3^. Then we did the hydrophilic treatment of the microneedle mold using oxygen plasma (at 1000 mTorr for 1 min, Plasma Cleaner, PDC-002, Harrick Plasma, Ithaca, NY, USA). We put the microneedle mold in a vacuum chamber to facilitate the solution deposition in the following step. After that, we dropped 300 μL of silk fibroin solution with insulin on the microneedle mold. We put the mold into a chamber with silica gel as a desiccant for deposition. After the solution was fully dried, we put the microneedle mold in a vacuum chamber again to facilitate the deposition of OSTE later. We then dropped 1.0 g OSTE on the microneedle mold and made it cure at room temperature for 24 h. When it was fully cured, we removed OSTE with dried silk fibroin from the microneedle mold. Figure 1 shows the detailed fabrication procedures.

### 2.2. Weight Ratio of Silk Fibroin

When adjusting the weight ratio of silk fibroin, we kept the overall volume and every other component the same. Specifically, when we prepared the silk fibroin solutions with 3.27%, 6.54%, 12.28%, and 19.62% silk fibroin, we used 200 μL of insulin solution, 300 μL of DI water, and 50 μL of 1% food dye solution, but with 0.0186 g, 0.0385 g, 0.0770 g, and 0.1343 g of silk fibroin, respectively.

### 2.3. Insertion of Microneedle Array Patches into Gels

We used gelatin gel (10 wt%) as a tissue model for microneedle insertion and drug diffusion studies as they are optically transparent and similar to human skin [19,20]. By preparing them, we placed the gelatin solution in the refrigerator to solidify and form gels. We pressed the microneedle array patches containing blue food dye against the surface of the gelatin gel to insert the needles into it. We recorded the process of microneedle insertion and drug release using a stereomicroscope.

## 3. Results and Discussion

### 3.1. Microneedle Forming

**Figure 2:**
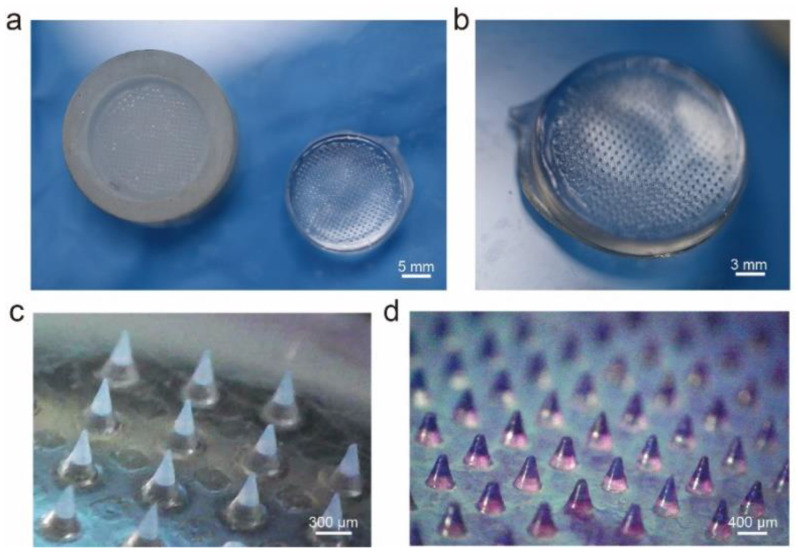
The pictures of (**a**) the microneedle mold and microneedle array patch, (**b**) a homogeneous microneedle array on the microneedle patch, (**c**) the microneedles with 0.01% purple food dye, and (**d**) the microneedles with 0.1% purple food dye under a stereomicroscope.

When OSTE is fully cured, it is easy to demold the microneedle array patch; the microneedle mold and microneedle array patch after demolding are shown in Figure 2a. We can see that a homogeneous microneedle array is formed, as shown in Figure 2b. The slender and tapered microneedles consist of two parts: needle tips in white color and a transparent OSTE base, as shown in Figure 2c. The microneedle array exhibits the microneedles with a height of 500 μm and a diameter of 270 μm at the bottom, and the distance between the two needle tips is 700 μm. We can see that the uniform microstructure is fabricated without any observable change. To better visualize the silk tip, we added some purple food dye to the silk fibroin, and we can see from Figure 2d that the needle tips made of silk fibroin are obviously formed on the transparent OSTE base. We also used SEM to observe the microstructure of the microneedles (shown in Appendix A).

### 3.2. Ratio of Tip and Base

**Figure 3:**
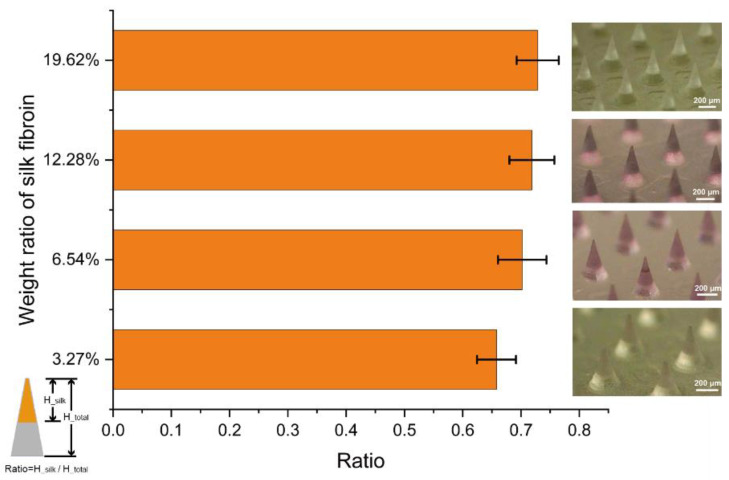
The change of the ratio of tip and base: the picture in the left shows the comparison of the ratio of silk fibroin tips between microneedle array patches with different weight ratios of silk fibroin. The pictures in the right are the microneedle arrays with the corresponding weight ratios of silk fibroin under a stereomicroscope, where color difference is due to the background difference.

To investigate the influence of the weight ratio of silk fibroin on the height of needle tips, we changed the weight ratio of silk fibroin from 3.27% to 19.62% and analyzed the ratio of the height of the silk fibroin tips to the height of the whole microneedles using ImageJ (http://rsb.info.nih.gov/ij/; accessed on 2 January 2022). We used a stereomicroscope to acquire the pictures of the microneedle array, where we kept placing the patches at 45° to the ground to maintain the consistency of analyses. We find that there is a very small difference between the ratio of the microneedles with different weight ratios of silk fibroin (shown in Figure 3), where the average ratio of tip and base is about 0.66, 0.70, 0.72, and 0.73, respectively. We suspect that silk tips formed with different silk fibroin concentrations may have different inner structures, such as more pores at low silk fibroin concentrations. However, more investigation is needed to be clear about the reason behind this.

### 3.3. Dummy Drug Release Experiments

**Figure 4:**
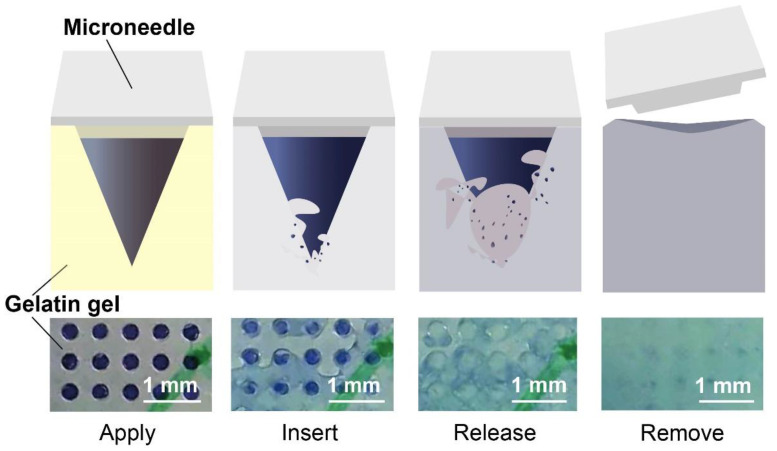
The process of application, insertion, release, and removal of microneedles. The pictures at the top show the schematic of the change of the tip. The pictures at the bottom show the change on the gelatin gel after inserting microneedles.

The process of applying microneedle array patches with the weight ratio of silk fibroin as 6.54% on the surface of gelatin gels, inserting microneedles into the gels, releasing drugs, and removing the patches is shown in Figure 4. For the first three pictures at the bottom, they were acquired from the bottom of the patches. The last one shows the needle holes with blue dye left on the surface of the gels after removing the patches. We removed the patches 20 min after we inserted them into gels. Further investigation of the concentration of the drug released can be performed by using spectroscopy at a specific wavelength, and the minimum force for successful microneedle insertion can be tested by using a force gauge [19,21].

## 4. Conclusions

We developed a facile method to fabricate microneedle array patches made of silk fibroin tips and a polymer OSTE base, and investigated the influence of the silk fibroin weight ratio on the length of the silk fibroin microneedle tip and their mechanical strength by inserting them into human-skin-mimicking gels. Such a microneedle array patch with composite structures can ensure that the whole silk tip penetrates and dissolves in the skin, with potential in increasing the drug delivery efficiency. Furthermore, rapid prototyping by polymer OSTE and cheap silk fibroins make it possible for large-scale manufacturing of such microneedle array patches.

## Figures and Tables

**Figure A1:**
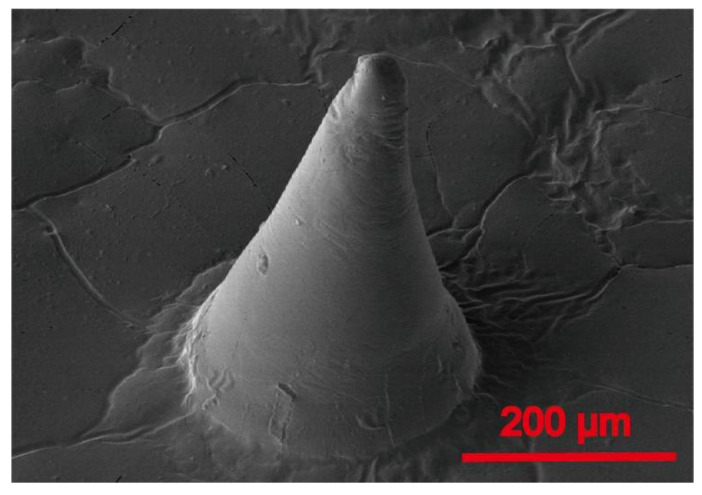
SEM picture of a microneedle.

## Data Availability

The data is available per request to guoweijin@stu.edu.cn.

## Appendix A. SEM Picture of Fabricated Microneedle

From the SEM (Regulus8100, Hitachi, Tokyo, Japan) picture of a microneedle shown in Figure A1, we can see the shape of the microneedle. Due to the gold coating, we cannot clearly see the distinction of OSTE and silk structure. In addition, we believe the high temperature and low pressure during gold coating may slightly deform the silk structure.
